# Using Silva pattern system to predict prognosis and plan treatment of invasive endocervical adenocarcinoma: a single-center retrospective analysis

**DOI:** 10.1186/s12905-022-02090-5

**Published:** 2022-12-02

**Authors:** Xiao Li, Shujie Pang, Yan Shen, Pengpeng Qu

**Affiliations:** 1 Department of Gynecological Oncology, Tianjin Central Hospital of Obstetrics and Gynecology, Tianjin Key Laboratory of Human Development and Reproductive Regulation, 156 Nankai Third Road, Nankai, Tianjin, 300100 People’s Republic of China; 2 Department of Pathology, Tianjin Central Hospital of Obstetrics and Gynecology, Tianjin Key Laboratory of Human Development and Reproductive Regulation, 156 Nankai Third Road, Nankai, Tianjin, 300100 People’s Republic of China

**Keywords:** Classification system, Invasive endocervical adenocarcinoma, Lymph node metastasis, Pattern-based, Risk stratification

## Abstract

**Background:**

This study evaluated the prognostic value of the Silva pattern system for invasive endocervical adenocarcinoma (EAC) by analysing its association with clinical and pathological features to provide more appropriate clinical management.

**Methods:**

A retrospective analysis including 63 patients with pathological diagnosis of invasive EAC was performed from March 2011 to December 2016 at our hospital. All pathological slides were reviewed by three senior pathologists, and cases were stratified into patterns A, B, or C by consensus according to the Silva pattern system criteria. Clinicopathological characteristics and follow-up of the three Silva subgroups were analysed.

**Results:**

Silva A, B, and C EAC patients were compared based on tumour size, clinical stage, lymphovascular invasion (LVI), and depth of invasion (DOI). The differences were found to be statistically significant (*p* < 0.01). There was no statistically significant difference in the proportion of lymph node metastasis among the three groups (*p* > 0.05) or in the recurrence and mortality rates of patients with Silva A, B, and C EAC (*p* > 0.05). Single factor analysis showed that tumour size, clinical stage, lymph node metastasis, LVI, and DOI were related to postoperative recurrence, whereas age, Silva classification, and postoperative recurrence were not correlated.

**Conclusion:**

The Silva classification system can predict lymph node status and prognosis of invasive EAC, but it cannot be used as an independent indicator. Individualized treatment plans should be adopted for patients with EAC.

## Background

Cervical cancer is the second most commonly diagnosed cancer and third leading cause of death due to cancer among women in less developed countries [1]. Endocervical adenocarcinoma (EAC) is the second most frequent cervical carcinoma following squamous cell carcinoma (SCC). Its relative and real incidence has been increasing in recent decades, accounting for 20–25% of all cervical cancers [2]. In addition, EAC is increasing in younger patients as compared to SCC, with EAC specifically increasing in patients of a reproductive age. The National Comprehensive Cancer Network (NCCN) guidelines determine how most patients with EAC at should be treated with radical surgery and nodal resection. These treatment modalities can cause surgery-related complications and affect fertility and childbearing capability. Therefore, differential treatment criteria appear to be needed for EAC, and objective standards need to be established as a basis for conservative treatment of EAC patients.

In recent years, a new pattern-based histopathologic classification for invasive EAC, named the Silva pattern system, has been proposed [3–6]. The Silva classification system categorizes EAC into 3 patterns on the basis of the morphologic features of the invasive carcinoma and is predictive of the risk for lymph node (LN) metastases. This study aimed to evaluate the prognostic value of the Silva pattern system for invasive EAC by analysing its association with clinical and pathological features to provide more appropriate clinical management.

## Methods

In 2011–2016, the incidence of invasive EAC in our hospital was approximately 9.7%, and the incidence of usual-type EAC was approximately 6.9%. A retrospective analysis of 63 patients with a pathological diagnosis of invasive EAC was performed from March 2011 to December 2016 in our hospital. Data analysed included patient age at diagnosis, International Federation of Gynecology and Obstetrics (FIGO) stage (using the FIGO 2018 Stage), histologic grade, surgical approach, postoperative complications, postoperative adjuvant therapy, depth of invasion (DOI), tumour size, lymphovascular invasion (LVI), LN metastasis, recurrence, and survival.

The selection criteria were as follows: (1) tumours diagnosed as invasive EAC, HPV-associated adenocarcinoma, usual type (as defined by the most recent World Health Organization classification (2014); (2) patients with tumours resected by cone/loop electrosurgical excision procedure or hysterectomy with tumour slides available for microscopic examination; (3) patients without preoperative chemotherapy; (4) lymphadenectomy with > 1 LN available for evaluation or clinical/radiologic evidence of metastatic LNs; and (5) complete case data to exclude patients with other tumours.

Patients with other unusual tumour types of EAC (adenosquamous, mucinous gastric/intestinal, adenoma malignum, serous, clear cell, and endometrioid), and patients with ≤ 1 resected LNs were excluded.

All pathological slides were reviewed by three senior pathologists, and cases were stratified into patterns A, B, or C by consensus according to the Silva pattern system criteria [3, 4, 6]. In brief, pattern A is defined by well-demarcated glands with the absence of single invasive cells or destructive stromal invasion, LVSI, and solid growth. Pattern B is characterized by early destructive stromal invasion arising from well-demarcated glands, with or without LVSI. Pattern C, which is associated with the worst prognosis, is characterized by diffuse destructive invasion or the presence of confluent epithelial growth (> 5 mm) or solid architecture. Cases with mixed patterns were classified based on the worst tumour pattern areas.

### Statistical analysis

SAS 9.4 was mainly used for statistical description and analysis of data. Measurement data are expressed as mean ± standard deviation/median ± quartile range, and count data are expressed as rates. According to the classification and distribution of variables, the Cochran-Mantel–Haenszel χ^2^ test and Fisher's exact probability method were used to compare the clinicopathological information of different subtypes of Silva; *p* < 0.05 was considered statistically significant. Taking the clinicopathological characteristics of patients as independent variables, two-level logistic regression was used to analyse the factors influencing cervical adenocarcinoma recurrence.

## Results

### Clinicopathological characteristics of patients with EAC (Table 1)

A total of 63 patients were included in the study. The average age was 47 ± 11 years (range, 23–73 years). The FIGO stage was IA in 11 patients (17.46%), IB in 47 patients (74.61%), IIA in 2 patients (3.17%), IIB in 2 patients (3.17%), and III in 1 patient (1.59%). Stage IA cases included 9 cases of stage IA1 and 2 cases of stage IA2. Stage IB patients included 20 patients with stage IB1, 19 patients with stage IB2, and 8 patients with stage IB3. Grade 1 was found in 23 (36.51%) tumours; grades 2 and 3 were found in 22 (34.92%) and 18 (28.57%) cases, respectively. Sixty-one patients underwent modified and radical hysterectomy or extrafascial hysterectomy, of which 53 patients underwent pelvic lymphadenectomy, 9 patients underwent cervical conization at first, and 2 patients received only cervical conization. LN metastasis was detected in 7 patients (11.11%) and LVI was identified in 21 patients (33.33%). The largest proportion of EAC patients (40%) had DOI of 3–5 mm. Regarding postoperative treatment, approximately 52% of patients received chemotherapy or a combination of radiotherapy and chemotherapy and five patients (7.94%) developed lymphatic cysts after surgery.

**Table 1:** Clinicopathological characteristics of patients with endocervical adenocarcinoma

Characteristics	N	%
*Age*		
20 ~	3	4.76
30 ~	11	17.46
40 ~	26	41.27
50 ~	20	31.75
60 ~	3	4.76
*Tumour size*		
< 2 cm	31	49.21
≥ 2 cm	32	50.79
*FIGO stage*		
IA	11	17.46
IB	47	74.61
IIA	2	3.17
IIB	2	3.17
III	1	1.59
*Grade*		
1	23	36.51
2	22	34.92
3	18	28.57
*Surgery*		
Hysterectomy	61	96.83
Cervical conization	2	3.17
*Lymphadenectomy*		
Yes	53	84.13
No	10	15.87
*LN metastasis*		
Yes	7	11.11
No	46	73.02
Unknown	10	15.87
*LVI*		
Yes	21	33.33
No	42	66.67
*DOI*		
< 3 mm	19	30.16
3–5 mm	25	39.68
≥ 5 mm	19	30.16
*Adjuvant therapy*		
Chemotherapy	21	33.33
Chemotherapy and radiation	12	19.05
Untreated	30	47.62

*DOI* Depth of invasion; *LN* Lymph node; *LVI* Lymphovascular invasion

### Clinicopathological characteristics of patients according to pattern classification

#### Pattern A

**Fig. 1:**
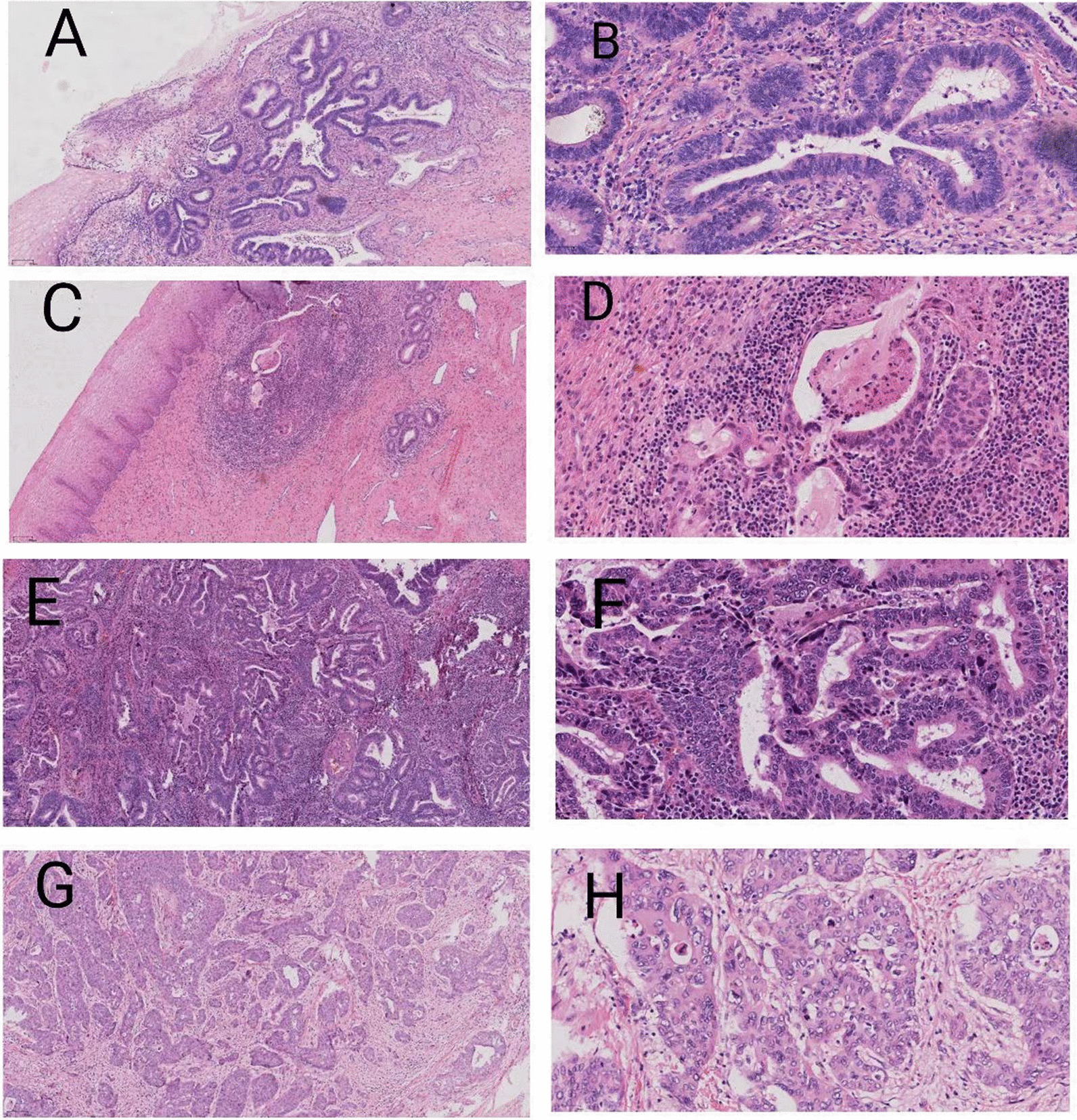
Pattern A: Characterized by well-demarcated glands without destructive stromal invasion or lymph-vascular invasion (**A**:H&E 10×,** B**:H&E 40×). Pattern B: Characterized by early destructive stromal invasion arising from pattern A glands (**C**:H&E 10×,** D**:H&E 40×). Pattern C: Characterized by diffusely infiltrative glands, with associated extensive desmoplastic response (**E**,**G**:H&E 10×,** F**,**H**:H&E 40×)

Eleven cases (17.5%, 11/63) contained morphologic features that corresponded to pattern A (Fig. 1A, B). All pattern A tumours were stage I. Among patients with pattern A tumours, 10 underwent modified and radical hysterectomy or extrafascial hysterectomy, and 6 of them had LNs removed. One patient underwent cervical conization. Nine patients had tumour size < 2 cm and 9 patients had DOI < 3 mm. Among 6 patients undergoing lymphadenectomy, 187 LNs were resected, with a range of 18–46 (mean 31.2) per patient. None of the patients had LVI or LN metastases (Table 2).

**Table 2:** Comparison of clinicopathological characteristics of endocervical adenocarcinoma in Silva classification

Characteristics	Silva A (N = 11)	Silva B (N = 9)	Silva C (N = 43)	Value	*p*-value*
Tumour size				χ^2^ = 7.974	**0.005**
< 2 cm	9	6	16		
≥ 2 cm	2	3	27		
FIGO stage				χ^2^ = 8.516	**0.004**
IA	7	2	2		
IB	4	6	37		
IIA	0	1	1		
IIB	0	0	2		
III	0	0	1		
Grade				χ^2^ = 18.769	** < 0.001**
1	10	4	9		
2	1	5	16		
3	0	0	18		
LN metastasis				–	**0.146**
Yes	0	0	7		
No	6	6	34		
LVI				–	** < 0.001**
Yes	0	0	21		
No	11	9	22		
DOI				χ^2^ = 6.224	**0.012**
< 3 mm	9	2	8		
3–5 mm	1	6	12		
≥ 5 mm	1	1	23		

The bold refers to the p value calculated by using statistical methods (Cochran-Mantel-Haenszel* χ*^2^ test and Fisher's exact probability method) to compare the clinicopathological information of different subtypes of Silva. *p* < 0.05 was considered statistically significant

^*^Fisher’s exact probability method

*DOI* Depth of invasion; *LN* Lymph node; *LVI* Lymphovascular invasion

#### Pattern B

Nine cases (14.3%, 9/63) had tumours with morphologic features that corresponded to pattern B (Fig. 1C, D), of which 8 patients had FIGO stage I tumours and 1 patient had stage IIA tumours. All patients underwent radical hysterectomy or extrafascial hysterectomy, including 6 cases of lymphadenectomy. Most of the cases had DOI 3–5 mm and tumour size < 2 cm. The total number of LNs resected was 185, with a range of 17–46 (mean 30.8) per patient. There were no LN metastases or LVI in any of the cases (Table 2).

#### Pattern C

Among 43 patients (68.2%, 43/63) with morphologic features of pattern C (Fig. 1E–H), 39 patients had stage I tumours, while 4 patients had stage II or higher tumours. Forty-two patients underwent radical hysterectomy or extrafascial hysterectomy, of which 41 underwent pelvic lymphadenectomy and only 1 underwent cervical conization. Twenty-seven patients had tumour size ≥ 2 cm, and 23 patients had DOI ≥ 5 mm. Overall, 1063 LNs were resected, with a range of 10–47 (mean 25.9) per patient, with only 21 positive LNs overall. A total of 21 cases demonstrated evidence of LVI. Seven patients showed LN metastases (6 IB, 1 III): 2 patients with 1 positive LN, 1 with 2 positive LNs, 1 with 3 positive LNs, 2 with 4 positive LNs and 1 with 6 positive LNs (Table 2).

Silva A, B, and C cervical adenocarcinoma patients were compared based on tumour size, clinical stage, histologic grade, LVI, DOI, and the differences were statistically significant (*p* < 0.01). There was no statistically significant difference in the proportion of LN metastasis between the three groups (*p* > 0.05) (Table 2).

### Follow-up results

All patients had follow-up data. Follow-up times ranged from 34 to 101 months. All patients with pattern A and B tumours were alive at the time of follow-up, with no evidence of recurrence. Five patients (11.6%) with pattern C tumours had recurrence and 2 patients died of the disease (4.7%). The recurrence rates (0, 0, 5/43) and mortality rates (0, 0, 2/43) of patients with Silva A, B, and C EAC were compared, and there was no significant difference (*p* > 0.05)(Table 3).

**Table 3:** Comparison of prognosis of endocervical adenocarcinoma in three types of Silva classification

Characteristics	Recurrence		Death	
	Yes	No	Yes	No
Silva A (N = 11)	0	11	0	11
Silva B (N = 9)	0	9	0	9
Silva C (N = 43)	5	38	2	41
*p*-value*	**0.475**		**0.462**	

The bold refers to the p value calculated by using statistical methods (Cochran-Mantel-Haenszel* χ*^2^ test and Fisher's exact probability method) to compare the clinicopathological information of different subtypes of Silva. *p* < 0.05 was considered statistically significant

^*^Fisher’s exact probability method

### Univariate logistic analysis of postoperative recurrence and related factors in patients with EAC

Single factor analysis was performed by taking the recurrence of postoperative cervical adenocarcinoma as the dependent variable and the correlation factors, such as age, tumour size, clinical stage, LN metastasis, LVI, DOI, and Silva classification, as independent variables. The results showed that tumour size, clinical stage, LN metastasis, LVI, and DOI were related to postoperative recurrence. The difference was statistically significant (*p* < 0.05). Age, Silva classification, and postoperative recurrence were not found to be correlated (*p* > 0.05) (Table 4).

**Table 4:** Univariate analysis results of influencing factors for endocervical adenocarcinoma recurrence

Characteristics	Recurrence		χ^2^	*p*-value*
	Present (N = 5)	Absent (N = 58)		
Age			6.213	**0.184**
20 ~	0	3		
30 ~	2	9		
40 ~	2	24		
50 ~	0	20		
60 ~	1	2		
Tumour size			5.261	**0.022**
< 2 cm	0	31		
≥ 2 cm	5	27		
FIGO stage			12.914	**0.012**
IA	0	11		
IB	4	42		
IIA	0	2		
IIB	0	2		
III	1	0		
LN metastasis			10.545	**0.001**
Yes	3	4		
No	2	44		
LVI			5.322	**0.021**
Yes	4	41		
No	1	17		
DOI			8.255	**0.016**
< 3 mm	0	19		
3–5 mm	5	20		
≥ 5 mm	0	19		
SILVA			–	**0.475**
A	0	11		
B	0	9		
C	5	38		

The bold refers to the p value calculated by using statistical methods (Cochran-Mantel-Haenszel* χ*^2^ test and Fisher's exact probability method) to compare the clinicopathological information of different subtypes of Silva. *p* < 0.05 was considered statistically significant

^*^Fisher’s exact probability method

*DOI* Depth of invasion; *LN* Lymph node; *LVI* Lymphovascular invasion

### Multivariate logistic analysis of postoperative recurrence and related factors in patients with EAC

Five univariate variables of cervical adenocarcinoma recurrence were simultaneously introduced into the unconditional logistic regression analysis model, and the Enter method was used to screen for the influencing factors. The results showed that tumour size, clinical stage, LN metastasis, LVI, and DOI were not related to the recurrence of cervical adenocarcinoma (*p* > 0.05) (Table 5). The stepwise regression method was used to screen the five variables, and the risk factors for postoperative recurrence of cervical adenocarcinoma (odds ratio > 1) were screened out. The results showed that LN metastasis was an independent predictor of recurrence (Table 6).

**Table 5:** Multivariate logistic analysis results of factors affecting cervical adenocarcinoma recurrence (Enter method)

Characteristics	β	SE	Wald	*p*-value	OR	95% CI
*Tumor size*						
< 2 cm	− 18.154	7604.496	< 0.001	0.998	< 0.001	–
≥ 2 cm	Ref					
*FIGO stage*						
IA	− 3.271	48,532.938	< 0.001	1.000	0.038	–
IB	− 21.203	40,192.950	< 0.001	1.000	< 0.001	–
IIA	− 39.470	47,973.810	< 0.001	0.999	< 0.001	–
IIB	− 39.813	47,112.250	< 0.001	0.999	< 0.001	–
III	Ref					
*LN metastasis*						
No	− 1.609	1.483	1.177	0.278	0.200	0.011–3.661
Yes	Ref					
*LVI*						
No	− 0.470	1.525	0.095	0.758	0.625	0.031–12.410
Yes	Ref					
*DOI*						
< 3 mm	− 18.901	11,985.524	< 0.001	0.999	< 0.001	–
3–5 mm	− 17.989	8494.734	< 0.001	0.998	< 0.001	–
≥ 5 mm	Ref					

*CI* Confidence interval; *DOI* Depth of invasion; *LN* Lymph node; *LVI* Lymphovascular invasion; *OR* Odds ratio; *SE* Standard error

**Table 6:** Multivariate logistic analysis results of factors affecting cervical adenocarcinoma recurrence (Stepwise method)

Characteristics	β	SE	Wald	*p*-value	OR	95% CI
LN metastasis	2.803	1.052	7.105	0.008	16.500	2.100–129.625

*CI* Confidence interval; *LN* Lymph node; *OR* Odds ratio; *SE* Standard error

### Comparison of clinicopathological characteristics and prognosis of patients with subgroups of Silva C

We further divided pattern C tumours into subgroups based on LVI and LN status. The first group included cases with identified LVI and documented LN metastases (LVI+/LN+) and was composed of 7 cases. The second group was composed of cases with identified LVI, but negative LNs (LVI+/LN−) and included 14 cases. The last group was composed of cases that lacked LVI and had negative LNs (LVI−/LN−) and included 20 cases. Their characteristics are listed in Table 7. There was no statistically significant difference in the clinicopathological characteristics of patients with different types of Silva C EAC (*p* ≥ 0.05). Of the 5 relapsed patients with pattern C tumours, 3 patients had LN metastases and LVI was present in 4 cases. Among the pattern C tumour subtypes, there were 3 relapse and 1 death in the LVI+/LN+group, 1 recurrence in the LVI+/LN− group, and 1 recurrence and 1 death in the LVI−/LN− group. There was no statistically significant difference in recurrence rate and mortality between the three subgroups (*p* ≥ 0.05) (Table 8).

**Table 7:** Characteristics of pattern C tumors based on LVI and LN status

Characteristics	Silva pattern C tumors			*p*-value
	LVI(+) LN(+)	LVI(+) LN(−)	LVI(−) LN(−)	
*Age*				**0.388**
20 ~	0	2	1	
30 ~	2	3	2	
40 ~	1	5	9	
50 ~	2	4	7	
60 ~	2	0	1	
*Tumor size*				**0.609**
< 2 cm	1	5	8	
≥ 2 cm	6	9	12	
*FIGO stage*				**0.605**
IA	0	0	0	
IB	6	13	18	
IIA	0	0	1	
IIB	0	1	1	
III	1	0	0	
*DOI*				**0.339**
< 3 mm	1	2	8	
3–5 mm	0	4	9	
≥ 5 mm	6	8	3	
Total	**7**	**14**	**20**	

The bold refers to the p value calculated by using statistical methods (Cochran-Mantel-Haenszel* χ*^2^ test and Fisher's exact probability method) to compare the clinicopathological information of different subtypes of Silva. *p* < 0.05 was considered statistically significant

*DOI* Depth of invasion; *LN* Lymph node; *LVI* Lymphovascular invasion

**Table 8:** Prognosis in three subgroups of pattern C tumors

Characteristics	Recurrence		Death	
	Yes	No	Yes	No
LVI(+) LN(+)	3	4	1	6
LVI(+) LN(−)	1	13	0	14
LVI(−) LN(−)	1	19	1	19
*p*-value	**0.050**		**0.427**	

The bold refers to the p value calculated by using statistical methods (Cochran-Mantel-Haenszel* χ*^2^ test and Fisher's exact probability method) to compare the clinicopathological information of different subtypes of Silva.* p* < 0.05 was considered statistically significant

*LN* Lymph node; *LVI* Lymphovascular invasion

## Discussion

Our research showed that there was a significant correlation between Silva classification and some adverse prognostic factors of cervical adenocarcinoma, such as tumour size, clinical stage, histologic grade, LVI, and DOI, indicating that Silva classification can be used to predict the prognosis of EAC. However, there was no statistically significant difference in the proportion of LN metastasis, recurrence rate, and mortality among patients with Silva A, B, and C EAC. Single factor analysis showed that there was no correlation between Silva classification and postoperative recurrence, while tumour size, clinical stage, LN metastasis, LVI, and DOI were associated with postoperative recurrence. LN metastasis is an independent predictor of recurrence. Therefore, our current data analysis showed that the Silva classification cannot be used alone as a guideline for treatment and prognosis, but should be combined with the patient’s clinical stage and high-risk factors.

The clinical treatment of EAC is mainly determined according to the FIGO clinical staging and NCCN guidelines. Multiple treatment modalities for cervical carcinoma include surgery, chemotherapy, radiation, and immunotherapy [7]. Because the prognosis of adenocarcinoma is worse than that of SCC, the treatment of EAC is more aggressive. Most patients undergo radical hysterectomy and pelvic lymphadenectomy, resulting in increased postoperative complications, such as lymphoedema and infertility in young women. Many patients did not have LN metastasis after surgery. EAC often has different clinical prognoses for patients in the same stage. At the same time, the pathological types of EAC and the degree of pathological differentiation have limited prognostic indicators. Most cervical adenocarcinomas are well-differentiated and moderately differentiated, and some very well-differentiated adenocarcinomas, such as minimal deviation adenocarcinoma, have a highly invasive biological behavior. Therefore, we need to find different ways to treat these patients.

The current staging method for EAC only considers the size of the tumour, and the DOI and does not consider the manner in which the tumour grows. The Silva pattern system is a risk stratification system based on the growth mode of tumour cells, as viewed under a microscope. Compared with FIGO staging, it can better evaluate and predict the biological behaviour of tumours and patient prognosis, thus providing a more reasonable treatment plan.

The Silva pattern-based system was proposed in 2013 and cases of EACs were classified into pattern A, pattern B, and pattern C according to this new system [3]. The Silva system was validated in subsequent studies, and the evaluation of its observers was also consistent [5, 6, 8, 9]. This risk stratification system better predicts the risk of LN metastasis and recurrence.

Pattern A tumors are usually identified under the microscope and there are no obvious clinical masses. There is no LVI in pattern A. In general, Stage IA1 lesions are considered to have an excellent prognosis and are treated via extrafascial hysterectomy or cervical conisation alone if preservation of fertility is desired. Even if the LNs are negative in most cases, radical hysterectomy and LN dissection (with or without para-aorta in the pelvis) are performed for stage IA2 and above lesions [10]. However, the complications of radical surgery and LN resection are high, including early complications such as bleeding, hematoma, pain, pyrexia, urinary tract infection, dysuria, and nerve damage. Late complications include urinary incontinence, dysuria, pelvic organ prolapse, venous thrombosis, lymphedema, and lymphatic cysts. In addition, these young patients lose fertility [11–13]. It is reported in the literature that patients with early-stage tumors rarely have evidence of LN metastasis and 0.8% of patients with stage IA1 and 1.7% of patients with stage IA2 have LN metastasis [14]. However, Ceballos and colleagues reported that a lymph node dissection complication rate of early EAC (IA1 and IA2) and negative LNs was 9% [15, 16], thus stressing the importance of better discriminating patients at lower risk for metastasis to spare them from unnecessary LN resection. According to relevant literature reports [4–6], tumours with pattern A had no LN metastasis and recurrence, and all patients were alive. In this study, 11 patients with pattern A tumours had stage I disease and had no LN metastasis and no LVI. Moreover, patients with pattern A tumours had no recurrence, and all of them survived. These results were similar to those of other studies [3, 6, 8, 17]. Therefore, conservative treatment is recommended for patients with pattern A. If the pattern A tumour persists in the cone and the margins are negative, the uterus is preserved, and lymphadenectomy is avoided. Bogani et al. [18] reported the margin status influenced the risk of developing cervical persistent/recurrent disease. So if the tumour involves the surgical margins of the cone, second cervical conization or wider excision can be necessary. In this study, 17.5% of the cases diagnosed as invasive EAC were classified as pattern A, meaning that nearly one-fifth of patients could potentially be spared from radical cervical cancer surgery and LN resection.

When the growth depth is shallower, the pathologist will differ in the diagnosis of EAC in situ or invasive EAC. The distinction between EAC in situ and early EAC is difficult, and a small portion of pattern A tumours may be interpreted by some pathologists as adenocarcinoma in situ (AIS). While studies indicate that a clear distinction of invasive EAC from AIS is not possible in up to 20% of cases, [19, 20] The Silva pattern system makes distinction in difficult cases irrelevant, as patients could be treated similarly, and will have an excellent prognosis.

Pattern B is characterized by localized destructive stromal invasion, mostly arising from neoplastic glands with a pattern A–like configuration with or without LVI. Patients with pattern B tumours, who all have stage I, rarely show nodal metastasis (only if there is LVI) and recurrences are very rare. Roma [6] reported that only 4 of 90 (< 5%) pattern B cases had LN metastasis, and all 4 had LVI. All patients had clinical stage I tumours, and only 1 patient experienced a vaginal recurrence that might be contamination related, similar to what has been proposed as the mechanism for vaginal recurrence in endometrial adenocarcinoma [21]. Our study showed that approximately 90% of patients had FIGO stage I tumours and there were no LN metastases or LVI in all cases. Because patients with pattern B tumours rarely presented with metastatic LNs, sentinel LN sampling in these patients might be beneficial in avoiding unnecessary morbidity of extensive LN dissection in these patients. Therefore, if a tumour in the cervical conization specimen revealed pattern B, then the treatment would include hysterectomy or conservative surgery plus sentinel LN sampling.

Pattern C tumours are by far the most aggressive and are defined by extensive desmoplastic and destructive reactions. Diaz De Vivar et al. [3] reported that 23.8% of patients had metastatic LNs, whereas 22.1% of patients eventually recurred. Roma et al. [4] reported that 16.9% of patients with pattern C presented with stage II or higher tumours. Further, 61.9% patients showed evidence of LVI, and almost 1 in 4 had metastatic LNs. Recurrence was reported in 21.5% of the patients. In line with the published literature, our study showed that LVI was present in 48.8% of cases, LN metastases were recorded in 17.1% of patients, and 11.6% suffered recurrences. Therefore, for the patients with pattern C tumours, aggressive treatment is justified. If the cervical conization reveals a pattern C tumour, a radical hysterectomy plus pelvic lymphadenectomy would be an appropriate treatment. In our study, we further divided pattern C tumours into subgroups based on LVI and LN status. The results showed that there was no statistically significant difference in the clinicopathological characteristics, recurrence rate, and mortality of patients with different types of Silva C cervical adenocarcinoma. Therefore, active treatment of patients with Silva C to prevent recurrence and closer follow-up may be beneficial regardless of the status of LVI and LNs.

In this study of 63 patients with cervical adenocarcinoma, Silva A type accounted for 17.5%, Silva B type accounted for 14.3%, and Silva C type accounted for 68.2%. If a different surgical method is selected based on this classification, it can allow nearly one-fifth of patients to retain fertility, and about 30% of patients will be free from systemic lymphadenectomy, thus avoiding the risks of associated complications and improving the quality of life after surgery.

Stolnicu et al. [22] confirmed that clinical outcomes differ between stage IA and IB1 endocervical adenocarcinomas and among substages, while multivariate analysis showed that Silva pattern and lymphovascular invasion are both significantly associated, in surgical cases, with clinical outcomes; these are the most important prognostic parametersamong low stage (IA1-IB1) endocervical adenocarcinomas. Therefore, future iterations of the FIGO staging should strongly consider including Silva pattern of invasion in the staging system.

There are some limitations to this study that need to be improved. Firstly, this was a single-centre study, the number of included cases was limited, and postoperative follow-up time was relatively short, resulting in some statistical data differences not being significant. Future studies should include more cases with a multicentre study. The second limitation of the study is its retrospective design. In the future, we plan to conduct prospective research data. Thirdly, the study only included EAC, the usual type and did not include special types: clear cell or serous carcinoma, mucinous adenocarcinoma, and so on.

## Conclusions

The Silva classification system can predict LN status and prognosis of invasive EAC, but it cannot be used as an independent indicator. Patients with pattern A EAC do not develop LN metastasis and therefore do not require LN resection. They have an excellent prognosis. Patients with pattern B rarely present with LN metastasis and/or recurrence, and the treatment may be using sentinel LN sampling. However, those with pattern C require aggressive treatment because of high rates of LN metastasis and recurrence. Complete lymphadenectomy should be considered for these patients, and adjuvant treatment might be justified on the basis of stage. In short, individualized treatment plans should be adopted for patients with EAC. To conclude, the Silva classification system can be used to choose patients who can safely undergo conservative treatment to preserve fertility and reduce post-treatment complications.

## Data Availability

All data generated or analysed during this study are included in this published article.

## Abbreviations

AIS: Adenocarcinoma in situ
CI: Confidence interval
DOI: Depth of invasion
EAC: Endocervical adenocarcinoma
FIGO: International federation of gynecology and obstetrics
LN: Lymph node
LVI: Lymphovascular invasion
NCCN: The national comprehensive cancer network
OR: Odds ratio
SE: Standard error
SCC: Squamous cell carcinoma

## References

[1] Torre LA, Bray F, Siegel RL, Ferlay J, Lortet-Tieulent J, Jemal A (2015). Global cancer statistics, 2012. CA Cancer J Clin.

[2] Fujiwara H, Yokota H, Monk B, Treilleux I, Devouassoux-Shisheboran M, Davis A (2014). Gynecologic cancer InterGroup (GCIG) consensus review for cervical adenocarcinoma. Int J Gynecol Cancer.

[3] De Vivar AD, Roma AA, Park KJ, Alvarado-Cabrero I, Rasty G, Chanona-Vilchis JG (2013). Invasive endocervical adenocarcinoma: proposal for a new pattern-based classification system with significant clinical implications: a multi-institutional study. Int J Gynecol Pathol.

[4] Roma AA, Mistretta TA, De Vivar AD, Park KJ, Alvarado-Cabrero I, Rasty G (2016). New pattern-based personalized risk stratification system for endocervical adenocarcinoma with important clinical implications and surgical outcome. Gynecol Oncol.

[5] Rutgers JK, Roma AA, Park KJ, Zaino RJ, Johnson A, Alvarado I (2016). Pattern classification of endocervical adenocarcinoma: reproducibility and review of criteria. Mod Pathol.

[6] Roma AA, De Vivar AD, Park KJ, Alvarado-Cabrero I, Rasty G, Chanona-Vilchis JG (2015). Invasive endocervical adenocarcinoma: a new pattern-based classification system with important clinical significance. Am J Surg Pathol.

[7] Di Tucci C, Schiavi MC, Faiano P, D'Oria O, Prata G, Sciuga V (2018). Therapeutic vaccines and immune checkpoints inhibition options for gynecological cancers. Crit Rev Oncol Hematol.

[8] Paquette C, Jeffus SK, Quick CM, Conaway MR, Stoler MH, Atkins KA (2015). Interobserver variability in the application of a proposed histologic subclassification of endocervical adenocarcinoma. Am J Surg Pathol.

[9] Alvarado-Cabrero I, Roma AA, Park KJ, Rutgers JKL, Silva EG (2017). Factors predicting pelvic lymph node metastasis, relapse, and disease outcome in pattern C endocervical adenocarcinomas. Int J Gynecol Pathol.

[10] National comprehensive cancer network: NCCN clinical practice guidelines in oncology (NCCN guidelines). Version 1. 2022. https://www.nccn.org.

[11] Zikan M, Fischerova D, Pinkavova I, Slama J, Weinberger V, Dusek L (2015). A prospective study examining the incidence of asymptomatic and symptomatic lymphoceles following lymphadenectomy in patients with gynecological cancer. Gynecol Oncol.

[12] Conte M, Panici PB, Guariglia L, Scambia G, Greggi S, Mancuso S (1990). Pelvic lymphocele following radical para-aortic and pelvic lymphadenectomy for cervical carcinoma: incidence rate and percutaneous management. Obstet Gynecol.

[13] Mehra G, Weekes A, Vantrappen P, Visvanathan D, Jeyarajah A (2010). Laparoscopic assisted radical vaginal hysterectomy for cervical carcinoma: morbidity and long-term follow-up. Eur J Surg Oncol.

[14] Poynor EA, Marshall D, Sonoda Y, Slomovitz BM, Barakat RR, Soslow RA (2006). Clinicopathologic features of early adenocarcinoma of the cervix initially managed with cervical conization. Gynecol Oncol.

[15] Ceballos K, Onuma K, Hauspy J, Rubabaza P, Rajagopalan A, Shaw D, Daya D (2012). Early invasive cervical adenocarcinoma: Is radical treatment indicated? [Abstract]. Lab Invest.

[16] Ceballos KM, Shaw D, Daya D (2006). Microinvasive cervical adenocarcinoma (FIGO stage 1A tumors): results of surgical staging and outcome analysis. Am J Surg Pathol.

[17] Byun JM, Cho HJ, Park HY, Kim YN, Lee KB, Sung MS (2019). Clinical significance of the pattern-based classification in endocervical adenocarcinoma, usual and variants. Int J Clin Oncol.

[18] Bogani G, Lalli L, Sopracordevole F, Ciavattini A, Ghelardi A, Simoncini T (2022). Development of a nomogram predicting the risk of persistence/recurrence of cervical dysplasia. Vaccines (Basel).

[19] Bean SM, Kurtycz DF, Colgan TJ (2011). Recent developments in defining microinvasive and early invasive carcinoma of the uterine cervix. J Low Genit Tract Dis.

[20] Ostör AG (2000). Early invasive adenocarcinoma of the uterine cervix. Int J Gynecol Pathol.

[21] Moschiano EJ, Barbuto DA, Walsh C, Singh K, Euscher ED, Roma AA (2014). Risk factors for recurrence and prognosis of low-grade endometrial adenocarcinoma; vaginal versus other sites. Int J Gynecol Pathol.

[22] Stolnicu S, Hoang L, Almadani N, De Brot L, Baiocchi G, Bovolim G (2022). Clinical correlation of lymphovascular invasion and Silva pattern of invasion in early-stage endocervical adenocarcinoma: proposed binary Silva classification system. Pathol.

